# Leg lymphedema caused by iliopectineal bursitis associated with destruction of a rheumatoid hip joint: A case report

**DOI:** 10.3892/etm.2013.1243

**Published:** 2013-08-02

**Authors:** GEN KUROYANAGI, KUNIO YAMADA, TSUKASA IMAIZUMI, JUN MIZUTANI, IKUO WADA, OSAMU KOZAWA, HARUHIKO TOKUDA, TAKANOBU OTSUKA

**Affiliations:** 1 Department of Orthopedic Surgery, Nagoya City University Graduate School of Medical Sciences, Nagoya, Aichi 467-8601;; 2 Department of Pharmacology, Gifu University Graduate School of Medicine, Gifu 501-1194;; 3 Department of Orthopedic Surgery, Komaki City Hospital, Komaki, Aichi 485-8520;; 4 Department of Clinical Laboratory, National Center for Geriatrics and Gerontology, Obu, Aichi 474-8511, Japan

**Keywords:** iliopectineal bursa, rheumatoid arthritis, total hip arthroplasty, rheumatoid hip joint, leg lymphedema

## Abstract

The present study describes a case of leg lymphedema due to iliopectineal bursitis associated with rheumatoid arthritis (RA), which was satisfactorily controlled by surgery and combination therapy with methotrexate (MTX) and tacrolimus. A 68-year-old male, who had a six-year history of RA, developed an iliopectineal bursa associated with destruction of the hip joint. The mass gradually increased in size, and there was swelling in his right lower extremity. The patient was subsequently hospitalized with increasing right hip pain and leg edema. A colorless transparent lymph fluid leaked from his leg, and leg lymphedema was thus diagnosed. The patient also had a 20-year history of myelodysplastic syndrome. Therefore, the extensive or total resection of the bursa was considered to be too invasive, so a partial bursal excision was performed via an anterior approach. Following the partial bursal excision, total hip arthroplasty (THA) was performed using the Hardinge approach. The leg lymphedema disappeared following the surgery, and the iliopectineal bursa was no longer enlarged. MTX and tacrolimus were postoperatively administered to strictly control the RA. The RA was subsequently well controlled, without any increases in the levels of inflammatory markers, such as C-reactive protein and matrix metalloproteinase-3. This case demonstrated that iliopectineal bursitis was resolved following THA, without complete excision of the intrapelvic bursa, and that strict RA control led to a good clinical course without recurrent inflammation of the bursa. Similar procedures may be beneficial in other patients contraindicated for resection of the entire bursa.

## Introduction

The iliopectineal bursa, which is a physiological structure of the iliofemoral joint in humans, reportedly causes pelvic or inguinal masses in rare cases (1). An enlargement of the iliopectineal bursa is often associated with rheumatoid arthritis (RA), osteoarthritis of the hip and pigmented villonodular synovitis.

RA is a chronic polyarthritis of unknown etiology, affecting ∼1% of the population globally (2). The clinical features of RA typically include polyarthritis with joint swelling of the hands and feet, although any of the large joints, such as the hips, knees, shoulders, elbows and ankles, may also become involved. Persistent synovitis results in bone destruction and various deformities of the joints (2). A variety of disease-modifying antirheumatic drugs (DMARDs) are available for the purpose of preventing joint destruction and improving the quality of life of patients with RA. Among them, methotrexate (MTX) has been the global standard DMARD, used either as a monotherapy or in combination therapy (3). MTX has been shown to improve the symptoms of RA and slow the radiographic progression of joint destruction (4).

Tacrolimus, another DMARD, targets T cells and causes the selective immunosuppression of T cells, the tacrolimus/tacrolimus-binding protein complex further binds to calcineurin to inhibit the translocation of cytoplasmic nuclear factors into the nucleus, thereby inhibiting the expression of cytokines, such as interleukin (IL)-2, IL-3, IL-4, interferon-γ and tumor necrosis factor (TNF)-α (5,6). It has been suggested that, in elderly patients with an insufficient response to DMARD therapy, tacrolimus is safe and well-tolerated and thus provides some clinical benefit (7). Another study indicated that tacrolimus may be successfully used as part of combination RA therapy with MTX (8). In addition, for a patient with a history of RA and myelodysplastic syndrome (MDS), combination therapy with tacrolimus and prednisolone improved the pancytopenia and the polyarthritis (9). Recently, certain biological agents have been shown to have significant efficacy in the treatment of RA (10), and novel biological agents continue to be developed.

Joint replacement is indicated when there is severe joint damage and an unsatisfactory control of symptoms with conservative treatment, such as medication or rehabilitation. The long-term outcomes of joint replacement are good, with only 4 to 13% of large joint replacements requiring revision within 10 years (11). The present study describes a case of leg lymphedema due to iliopectineal bursitis associated with RA, which was successfully controlled by surgical resection and combination therapy with MTX and tacrolimus. An ethics committee in Komaki City Hospital (Komaki, Japan) approved this study. Informed consent was obtained from the patient.

## Case report

A 68-year-old male with a six-year history of RA and a 20-year history of MDS was treated at Komaki City Hospital. To treat the patient’s MDS, metenolone was administered from 2005 to 2006, and for anemia, blood transfusions were performed as required. For the treatment of the patient’s RA, the patient first received bucillamine in 2006, prior to the bucillamine being replaced by salazosulfapyridine in January 2008. In October 2008, the patient complained of right hip joint pain following a fall. In March 2009, the patient became aware of a right inguinal soft tissue mass. The mass gradually increased in size and swelling was present in the right lower extremity. At that time, the patient was submitted to hospital with gradually increasing right hip pain and leg edema.

Upon physical examination, the patient was measured to be 150 cm tall and 44 kg in weight, with a body temperature of 36.6°C. The inguinal mass was easily palpable, but localized heat was not apparent around the hip. The range of motion (ROM) of the right hip was extremely limited. The ROM was 50° in flexion, 0° in extension, 20° in abduction, 20° in adduction, 20° in external rotation and 0° in internal rotation. The right leg of the patient was shorter than the left by 2 cm, and a diffuse swelling of the lower extremity was observed. A colorless transparent lymph fluid leaked from the patient’s leg, and leg lymphedema was thus diagnosed.

Hematological examination revealed a white blood cell (WBC) count of 6,800/*μ*l, a C-reactive protein (CRP) level of 11.0 mg/dl, a matrix metalloproteinase-3 (MMP-3) level of 209 ng/ml and a rheumatoid factor (RF) level of 106 IU/ml (Table I).

**Figure 1.**
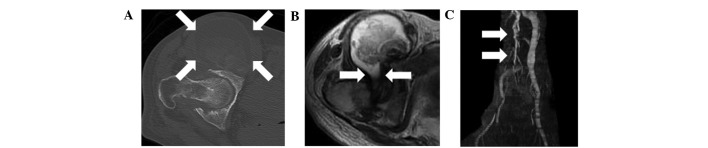
(A) Computed tomography (CT) showed an enlarged mass anterior to the right hip joint (white arrow). (B) Magnetic resonance imaging (MRI) showing an enlarged mass anterior to the right hip joint. An axial T2-weighted MRI image showed that there was a connection between the mass and the right hip joint (white arrow). (C) MRI venography demonstrated that the right femoral vein was displaced medially by the mass (white arrow).

Plain radiographs showed destruction of the right hip and collapse of the right femoral head. Computed tomography (CT) showed joint space narrowing and an enlarged mass anterior to the right hip joint (Fig. 1A). Magnetic resonance imaging (MRI) showed an enlarged mass anterior to the right hip joint. The mass displaced the iliopsoas muscle laterally, and was shown to connect with the joint space of the right hip (Fig. 1B). The signal intensity of the lesion was abnormal on T1- and T2-weighted images. An MRI venography showed that the femoral vein was displaced medially by the mass (Fig. 1C). Needle aspiration yielded 110 ml of black-brown fluid. The cytology and culture results were negative. The diagnosis of iliopectineal bursitis associated with destruction of a rheumatoid hip joint was made on the basis of these findings, and surgery was thus performed.

**Figure 2.**
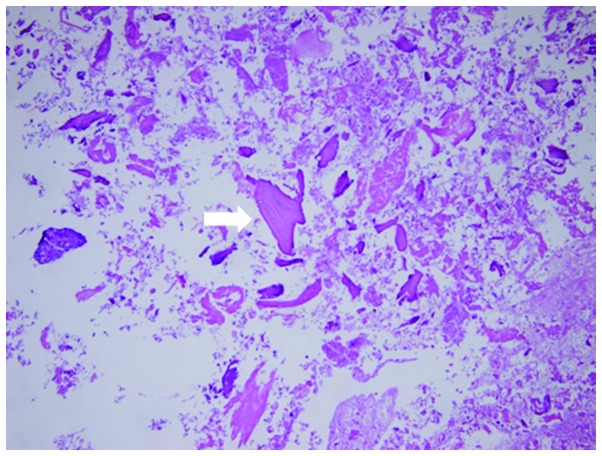
Histological appearance of the synovial fluid in the contents of the bursa (hematoxylin and eosin staining; magnification, ×100). Bone and cartilage debris and fibrinoid necrosis depositions were apparent (white arrow).

Surgical excision was carried out via an anterior approach. The cystic black-brown fluid with the fibrinoid necrotic tissue had erupted. The contents of the cyst were resected, and partial bursal excision was performed. Following the partial bursal excision, total hip arthroplasty (THA) was performed using the Hardinge approach. The contents of the bursa were stained with hematoxylin and eosin. The uptake of bone cartilage debris and fibrinoid necrosis deposition was apparent (Fig. 2). This content of the bursa has the same structure as the synovial tissue of the hip joint.

THA was performed with a desirable surgical result, and the RA disease activity was suppressed by the use of MTX at one month subsequent to the surgery. MTX was initiated at a dose of 4 mg postoperatively, and the dose subsequently remained unchanged. To control the RA more strictly, tacrolimus was added to the MTX six months subsequent to the surgery. Tacrolimus was initiated at a dose of 1.0 mg. The RA was well controlled, without any increases in the levels of inflammatory markers, such as CRP and MMP-3, being observed (Table I). The MDS control did not change postoperatively. The patient’s leg lymphedema disappeared rapidly following the surgery and the iliopectineal bursa did not become re-enlarged. The patient was able to walk normally without complaint one year subsequent to the surgery.

## Discussion

The iliopectineal bursa is the largest bursa in the human body (1). It lies posterior to the iliopsoas tendon, lateral to the femoral vessels and overlies the hip joint capsule. The size of the bursa normally ranges between 5 and 7 cm in length and 2 and 4 cm in width (1,12). Communication existing between the iliopectineal bursa and the hip joint has been demonstrated in ∼14% of cadavers (13,14). In the present case, the size of the bursa was 6 cm in length and 7 cm in width. An enlargement of the iliopectineal bursa was first described in 1834 by Fricke (15). An enlargement of the iliopectineal bursa is often associated with RA, osteoarthritis of the hip and pigmented villonodular synovitis. In addition, iliopectineal bursitis has been associated with acute destruction of the hip joint and rapid resorption of the femoral head in patients with RA (1,12,16,17,18).

Coventry *et al* discussed three possible mechanisms responsible for the occurrence of synovial cysts in patients with RA (19). Firstly, the overproduction of synovial fluid in a rheumatoid joint may increase the intra-articular pressure and distend the capsule in the joint. A second theory is that the involvement of the iliopectineal bursa in the rheumatoid process may lead to the formation of excessive quantities of fluid, enlargement of the bursa and hypertrophic and villous proliferation of the bursal lining. The third theory is that necrosis of a subcutaneous periarticular rheumatoid nodule may result in the formation of a juxta-articular cyst simulating the appearance of a synovial cyst.

In the present case, the overproduction of synovial fluid in the arthritic joint may have led to increased intra-articular pressure and protrusion of the synovial membranes into the potential space of the iliopectineal bursa, via communication between the bursa and the hip joint. The elevated pressure, due to fluid overproduction in the bursa, may have irritated the femoral vessels and exacerbated the leg lymphedema. When the iliopectineal bursa is enlarged, it compresses adjacent structures, such as the femoral vessels, the femoral nerve, the urinary tract and the bladder, and may cause a variety of symptoms (20). However, the leakage of a colorless transparent lymph fluid from the leg and lymphedema of the leg have, to the best of our knowledge, not been reported previously as complications of iliopectineal bursitis.

Matsumoto *et al* reported that they had not identified any incidences of iliopectineal bursitis recurring following THA, regardless of whether the patient had previously undergone a bursal excision (21). In the present case, we performed a partial bursal excision, due to the fact that the patient had a history of MDS, and extensive or total resection of the bursa was considered to be too invasive. The iliopectineal bursitis resolved following the THA, without complete excision of the intrapelvic bursa. If the RA is well controlled postoperatively, we propose that synovitis of the hip joint may be prevented, and that the production of synovial fluid in the hip joint, which is the likely cause of bursitis, may also be suppressed. Moreover, since communication between the hip joint and the bursa is one-way, due to a valve mechanism, we considered that the synovial fluid in the hip joint was not likely to spread to the bursa following THA.

In conclusion, we report the case of a 68-year-old male with RA who developed leg lymphedema due to an enlarged iliopectineal bursa associated with destruction of the hip joint. The iliopectineal bursitis was resolved following THA without complete excision of the intrapelvic bursa. The patient’s leg lymphedema disappeared quickly, and the iliopectineal bursa has not re-enlarged since the surgery. MTX and tacrolimus treatments were initiated following the surgery to provide RA control. Therefore, the present results strongly suggest that the iliopectineal bursitis was resolved following THA, without complete excision of the intrapelvic bursa, and that strict RA control led to a good clinical course without recurrent inflammation of the bursa.

## Figures and Tables

**Table I.** Laboratory data.

Parameters	At the time of hospitalization	Eighteen months after surgery	Normal range
Complete blood counts			
WBC (/*μ*l)	6800	6200	3500–9000
Segs (%)	85.0	91.0	
Stabs (%)	0.0	0.0	
Lymphocytes (%)	11.0	7.0	
Monocytes (%)	3.0	2.0	
Eosinophils (%)	0.0	0.0	
Basophils (%)	1.0	0.0	
Blast (%)	0.0	0.0	
RBC (/*μ*l)	410×10^4^	394×10^4^	410–530×10^4^
Hb (g/dl)	13.2	13.1	12.4–17.2
Hct (%)	39.6	39.3	38.0–54.0
Plt (/*μ*l)	23.8×10^4^	21.3×10^4^	14.0–35.0×10^4^
Blood Chemistry			
Total protein (g/dl)	6.4	6.0	6.7–8.3
Albumin (g/dl)	3.3	3.7	4.0–5.0
AST (IU/l)	34.1	31.5	13.0–33.0
ALT (IU/l)	30.2	29.5	6.0–30.0
Urinary nitrogen (mg/dl)	22.1	11.6	8.0–22.0
Creatinine (mg/dl)	0.67	0.87	0.60–1.10
Serum sodium (mEq/l)	139.0	140.6	138.0–146.0
Serum potassium (mEq/l)	4.0	3.9	3.6–4.9
Serum chloride (mEq/l)	104.0	107.0	99.0–109.0
Immunology			
CRP (mg/dl)	11.0	1.7	0.0–0.3
RF (IU/ml)	106.4	22.7	0.0–15.0
MMP-3 (ng/ml)	208.8	234.9	36.9–121.0

WBC, white blood cell; RBC, red blood cell; Hb, hemoglobin; Hct, hematocrit; Plt, platelet; AST, L-aspartate aminotransferase; ALT, L-alanine aminotransferase; CRP, C-reactive protein; RF, rheumatoid factor; MMP-3, matrix metalloproteinase-3.
